# MMDD-Ensemble: A Multimodal Data–Driven Ensemble Approach for Parkinson's Disease Detection

**DOI:** 10.3389/fnins.2021.754058

**Published:** 2021-11-01

**Authors:** Liaqat Ali, Zhiquan He, Wenming Cao, Hafiz Tayyab Rauf, Yakubu Imrana, Md Belal Bin Heyat

**Affiliations:** ^1^Department of Electrical Engineering, University of Science and Technology, Bannu, Pakistan; ^2^Guangdong Multimedia Information Service Engineering Technology Research Center, Shenzhen University, Shenzhen, China; ^3^Faculty of Engineering & Informatics, University of Bradford, Bradford, United Kingdom; ^4^School of Computer Science and Engineering, University of Electronic Science and Technology of China, Chengdu, China; ^5^School of Electronic Science and Engineering, University of Electronic Science and Technology of China, Chengdu, China

**Keywords:** blending, multimodal data processing, Parkinson's disease, support vector machine, voting based ensembles

## Abstract

Parkinson's disease (PD) is the second most common neurological disease having no specific medical test for its diagnosis. In this study, we consider PD detection based on multimodal voice data that was collected through two channels, i.e., Smart Phone (SP) and Acoustic Cardioid (AC). Four types of data modalities were collected through each channel, namely sustained phonation (P), speech (S), voiced (V), and unvoiced (U) modality. The contributions of this paper are twofold. First, it explores optimal data modality and features having better information about PD. Second, it proposes a MultiModal Data–Driven Ensemble (MMDD-Ensemble) approach for PD detection. The MMDD-Ensemble has two levels. At the first level, different base classifiers are developed that are driven by multimodal voice data. At the second level, the predictions of the base classifiers are fused using blending and voting methods. In order to validate the robustness of the propose method, six evaluation measures, namely accuracy, sensitivity, specificity, Matthews correlation coefficient (MCC), and area under the curve (AUC), are adopted. The proposed method outperformed the best results produced by optimal unimodal framework from both the key evaluation aspects, i.e., accuracy and AUC. Furthermore, the proposed method also outperformed other state-of-the-art ensemble models. Experimental results show that the proposed multimodal approach yields 96% accuracy, 100% sensitivity, 88.88% specificity, 0.914 of MCC, and 0.986 of AUC. These results are promising compared to the recently reported results for PD detection based on multimodal voice data.

## 1. Introduction

Parkinson's disease (PD) is a neurodegenerative disease of the central nervous system (CNS) effecting approximately 6.3 million populations worldwide across all genders, races, and cultures. It causes partial or complete loss of speech, motor reflexes, and behavioral and mental processes (Jankovic, 2008; Khorasani and Daliri, 2014; Ali et al., 2019b). In 1817, Dr. James Parkinson described and named the disease (Langston, 2002). Common symptoms of PD include tremor at rest, rigidity, bradykinesia, postural instability, visual problems, dementia, memory loss, and confusion, which manifest with thinking, judgment, and other features of cognitive function (Janghel et al., 2017). However, dysphonia (defective use of the voice), hypophonia (reduced volume), monotone (reduced pitch range), and dysarthria (difficulty with articulation of sounds or syllables) are important speech impairments found in People with Parkinsonism (PWP) (Sakar et al., 2013). Recently, PD detection through voice data has drawn significant attention owing to the following reasons. First, vocal impairments are hypothesized to be earliest symptoms of the disease (Duffy, 2013). Second, it is claimed that nearly 90% of PWP show voice impairments (Ho et al., 1999; Sakar et al., 2013). Third, PD detection based on voice data enables telediagnosis of the disease (Tsanas et al., 2012; Sakar et al., 2013; Ali et al., 2019a). Till now, there are no blood or laboratory tests to diagnose PD cases (Li et al., 2019). Therefore, automated learning system based on machine learning is required to provide an efficient way of evaluating the disease (Ravì et al., 2017).

In literature, different studies have been conducted for automated detection of PD based on voice and speech data (Das, 2010; Chen et al., 2013; Zuo et al., 2013; Behroozi and Sami, 2016; Benba et al., 2016, 2017; Gürüler, 2017; Cai et al., 2018; Ali et al., 2019c,d). Little et al. presented a method to analyze PD by measuring the dysphonia in vowel “a” phonation data from 31 subjects and obtained 91% accuracy (Little et al., 2009). Recently, Sarkar et al. performed a comparative study on different feature extraction methods for PD detection based on replicated vowel “a” phonation data and showed that tunable Q-factor wavelet transform (TQWT) and Mel-frequency cepstral possess complementary information about PD (Sakar et al., 2019). Vaiciukynas et al. collected multimodal voice and speech data for PD detection (Vaiciukynas et al., 2017). They collected four different modalities of data and extracted 18 different feature sets. They achieved PD detection performance of 79%. Almeida et al. utilized the multimodal voice and speech data collected in Vaiciukynas et al. (2017) and explored feasibility of different machine learning methods on the 18 extracted feature sets (Almeida et al., 2019). They obtained highest PD detection accuracy of 94%.

In recent years, multimodal learning and ensemble learning–based systems have gained significant attention owing to their improved performance (Gao et al., 2020c; Gheisari et al., 2021). Kassani et al. proposed multimodal sparse extreme learning machine (ELM) classifier for adolescent brain age prediction (Kassani et al., 2019). Their proposed multimodal sparse ELM method outperformed conventional ELM and sparse Bayesian learning ELM method in terms of classification accuracy. Luo et al. proposed multimodal neuroimaging (fMRI, DTI, sMRI) data-based prediction of attention-deficit/hyperactivity disorder (ADHD) (Luo et al., 2020). Their experimental results showed that bagging ensemble approach with SVM base classifiers produced promising results. Kumar et al. proposed a hypothesis that different architectures of convolutional neural networks (CNNs) learn different levels of semantic image representations (Kumar et al., 2016). Based on the hypothesis, they developed an ensemble of fine-tuned CNN for medical image classification. The ensemble approach outperformed other established CNNs. Poria et al. proposed multimodal approach for sentiment analysis (Poria et al., 2017). They utilized audio, video, textual data modalities. Their results showed that textual modality offered best accuracy of 79.14%, while fusion of the three modalities produced accuracy of 87.89%. Recently, Hao et al. proposed emotion recognition based on visual audio data (Hao et al., 2020). They proposed blending ensemble approach for the fusion of the audio and visual data for emotion recognition. Their proposed method outperformed many state-of-the-art methods.

**Figure 1:**
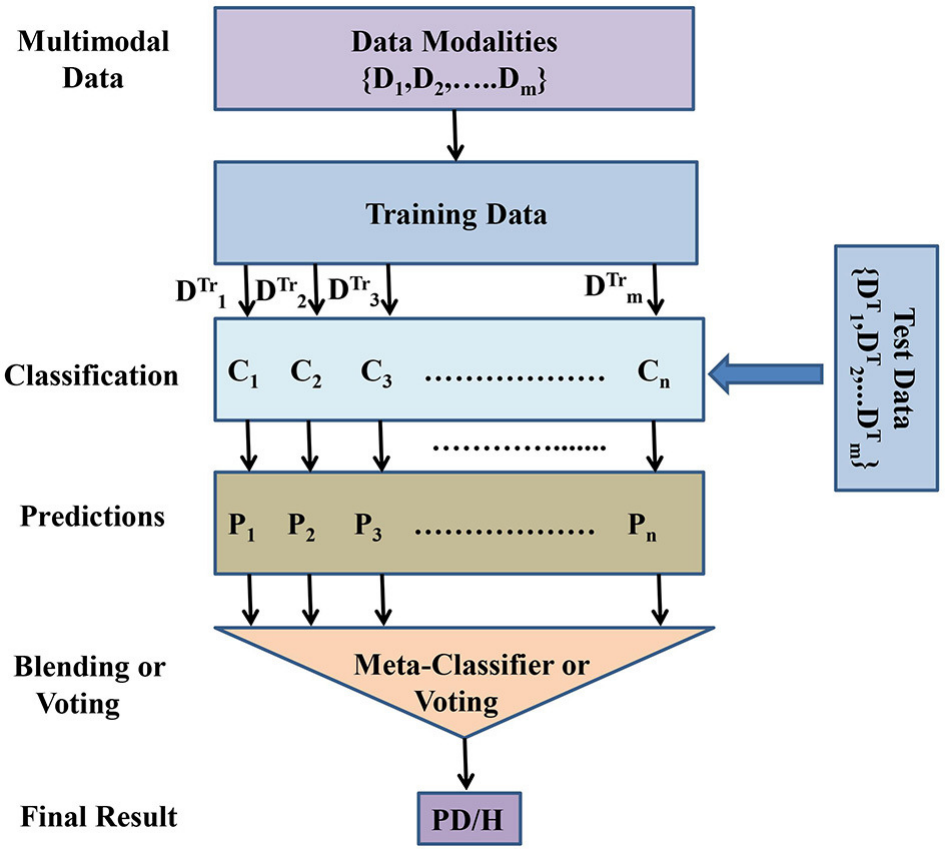
Block diagram of the proposed MultiModal Data–Driven Ensemble (MMDD-Ensemble) model. *D*_*i*_: The *i*th data modality. $D_{i}^{Tr}$: Training data of the *i*th data modality: $D_{i}^{T}$: Testing data of the *i*th data modality. *C*_*i*_: *i*th classification model. *P*_*i*_: Predicted output of the *i*th classification model. PD/H: Final prediction of the MMDD-Ensemble model, PD for PD patient, and H for healthy subject.

Motivated by the automated methods based on multimodal learning and ensemble learning, in this paper we also tried to explore feasibility of multimodal and ensemble learning for PD detection. This study deals with two research questions. The first question in case of PD detection based on multimodal data is what kind of features and data modality possess better information about PD. Second, how the multimodalities can be exploited to improve PD detection accuracy. Hence, this study has twofold contributions. (1) In this paper, we develop a number of machine learning models in order to explore the optimal data modality and features having complementary information about PD. (2) This paper proposes a MultiModal Data–Driven Ensemble (MMDD-Ensemble) approach for improved PD detection. The proposed MMDD-Ensemble has two levels. At the first level, different base classifiers are developed that are driven by different types of voice data (multimodalities). At the second level, the predictions of the base classifiers are fused using two different methods i.e., blending and voting. The working of the proposed MMDD-Ensemble approach is more clearly depicted in Figure 1.

The rest of the paper is organized as follows: In section 2, the details about the multimodal data and features are given. Moreover, the proposed method is also discussed in detail. In section 3, the evaluation measures are discussed, while section 4 discusses results. The last section is about conclusion of the study.

## 2. Materials and Methods

### 2.1. Multimodal Voice and Speech Data

The data used in this paper were collected by performing two vocal tasks, namely phonation and speech, which were treated as two separate modalities (Vaiciukynas et al., 2017). The speech modality data were collected from a phonatically balanced sentence in Lithuanian language “granny had a little greyish goat.” The data of phonation modality were obtained from voicing of vowel “a,” which was repeated 3 times. Two more modalities were also obtained for experiments by splitting the speech data into voiced and unvoiced modalities by utilizing Praat software. During the data collection process, two types of channels were utilized, i.e., Smart Phone (SP) and Acoustic Cardiod (AC). The microphones of both the channels were located at a distance of 10 cm from the subject's mouth.

From each type of modality, 18 different kinds of features are extracted; however, we considered 17 sets for experimentation in this study. Details of these different types of feature sets are given in Table 1. The feature sets numbered from 1 to 13 (except 6) in Table 1 are extracted using OpenSMILE toolkit (Eyben et al., 2013). The feature set numbered 6 in Table 1 was extracted using Essentia library (Bogdanov et al., 2013). Essentia is a well-known C++ library developed for the purpose of audio analysis. The feature set numbered 15 in Table 1 was extracted using a java-based library namely MPEG7AudioEnc (Crysandt et al., 2004).

**Table 1:** Features extraction methods and statistical analysis of the features.

**No**	**Fea. ext. method**	**Abbreviation**	**Tool/Study**
1	Avec2011	AV1	OpenSMILE toolkit Eyben et al. (2013)
2	Avec2013	AV2	OpenSMILE toolkit Eyben et al. (2013)
3	Emo_large	EL	OpenSMILE toolkit Eyben et al. (2013)
4	Emobase	EM1	OpenSMILE toolkit Eyben et al. (2013)
5	Emobase2010	EM2	OpenSMILE toolkit Eyben et al. (2013)
6	Essentia_descriptors	ED	Essentia Bogdanov et al. (2013)
7	IS09_emotion	IS1	OpenSMILE toolkit Eyben et al. (2013)
8	IS10_paraling	IS2	OpenSMILE toolkit Eyben et al. (2013)
9	IS10_paraling_compat	IS3	OpenSMILE toolkit Eyben et al. (2013)
10	IS11_speaker_state	IS4	OpenSMILE toolkit Eyben et al. (2013)
11	IS12_speaker_trait	IS5	OpenSMILE toolkit Eyben et al. (2013)
12	IS12_speaker_trait_compat	IS6	OpenSMILE toolkit Eyben et al. (2013)
13	IS13_ComPare	IS7	OpenSMILE toolkit Eyben et al. (2013)
14	jAudio_features	JA	jAudio McEnnis et al. (2006)
15	MPEG7_descriptors	MP	MPEG7AudioEnc Crysandt et al. (2004)
16	Tsanas	TS	Tsanas (2012)
17	YAAFE_features	YA	YAAFE toolbox Mathieu et al. (2010)

The jAudio features that are numbered 14 in Table 1 were extracted through a java-based library namely jAudio (McEnnis et al., 2006). The jAudio library was developed for standardized features extraction mainly for the purpose of music classification. The feature set numbered 17 in Table 1 is named YAAFE. It was extracted using YAAFE features extraction toolbox (Mathieu et al., 2010). Finally, a feature set based on time frequency measures was extracted and named Tsanas features (Tsanas, 2012).

### 2.2. Multimodal Data–Driven Ensemble Approach

In this study, two types of questions are considered and addressed. First, what kind of features and data modality possess better information about PD detection. Previous studies arrived at conflicting outcomes. Some studies pointed out that Essentia features provide better PD detection for AC channel (Vaiciukynas et al., 2017), while other studies concluded that YAAFE features yield better PD detection accuracy for the AC channel data (Almeida et al., 2019). Similarly, some studies pointed out that AC speech modality provides better PD detection (Vaiciukynas et al., 2017), while other concluded that AC phonation modality yields better PD detection (Almeida et al., 2019). After critically analyzing the results obtained in these studies, we arrived at the conclusion that the main reason of such conflicting results is that the previous methods presented conclusions based on just one kind of machine learning model. However, one model can be sensitive to one kind of feature set or data modality while another model can be sensitive to another kind of feature set or modality. Hence, a more pertinent solution is to utilize a number of machine learning models and then decide the optimal data modality and feature set based on the commutative results of the models. After exploring a range of machine learning models under the above discussed framework, we arrive at the conclusion that the highest or best PD detection accuracy is 88% under unimodal approach. The low rate of PD detection motivated the development of a new model that can exploit the benefit of multimodal data and produce better PD detection accuracy.

The second question that we addressed in this study was how the effects of multimodalities and multiple types of feature sets can be exploited to facilitate improved PD detection. Hence, we developed and evaluated an MMDD-Ensemble. In literature, two types of fusion methods are used for multimodal data. One approach is feature level fusion where the features of different data modalities are fused and one resultant feature vector is obtained (Wang et al., 2021). The second approach is decision level fusion where the multiple types of modalities are processed independently by machine learning models and the predictions, i.e., decisions are fused to arrive at final decision (Wang et al., 2021). In this study, we utilized decision level fusion. The proposed MMDD-Ensemble exploits the policies of blending and voting for fusing the decisions of multimodal data–driven base classifiers. The working of the proposed MMDD-Ensemble model is described and formulated as follows:

The proposed MMDD-Ensemble works in two levels. At the first level, base classifiers are developed by utilizing the multiple types of data modalities. Let *D* = {*D*_1_, *D*_2_, ...., *D*_*i*_, ........, *D*_*m*_} be a set of multimodal data, where *D*_*i*_ denotes *i*th data modality. For each data modality, a number of feature sets have been recorded denoted by *F* = {*F*_1_, *F*_2_, ...., *F*_*j*_, ........, *F*_*n*_}. In order to develop the proposed MMDD-Ensemble model, we discarded those modalities that yield poor prediction accuracy. As a result, we are left with *o* number of optimal data modalities. The data of these modalities are partitioned into training and testing datasets resulting in $D^{Tr}=\left\{D_{1}^{Tr},D_{2}^{Tr},D_{3}^{Tr},........,D_{o}^{Tr}\right\}$ and $D^{T}=\left\{D_{1}^{T},D_{2}^{T},D_{3}^{T},........,D_{o}^{T}\right\}$, where $D_{i}^{Tr}$ denotes the training dataset of the *i*th modality and $D_{i}^{T}$ denotes the testing dataset of the *i*th modality.

At the first level, i.e., base level, *p* number of base classifiers are developed. The set of *n* classifiers denoted by *C* = {*C*_1_, *C*_2_, ....., *C*_*k*_, ........, *C*_*p*_} is constructed such that *C*_*k*_ is trained and tested by using one specific type of data modality. That is each classifier is trained and tested by using the training and testing dataset of a specific modality. After training the level 1 classifiers (base classifiers), they are tested using the testing data. Thus, during the testing phase the base classifiers will yield a set of predictions denoted by $P^{T}=\left\{P_{1}^{T},P_{2}^{T},....,P_{k}^{T},........,P_{p}^{T}\right\}$, where $P_{k}^{T}$ is the prediction of the classifier *C*_*k*_.

At the second level (meta-level), we need to fuse the effects or predictions of the level 1 classifiers to arrive at the final prediction of the two level MMDD-Ensemble model. In this paper, we use two different criteria for the fusion of the predictions at level 2, namely, voting and blending. The voting approach is simple. During the training phase, the level 1 classifiers are trained using the training dataset. During the testing phase, the trained base classifiers are tested on the testing data resulting in level 1 predictions, i.e., $P^{T}=\left\{P_{1}^{T},P_{2}^{T},P_{3}^{T},........,P_{p}^{T}\right\}$. To evaluate the final prediction by fusing the level 1 prediction, a majority voting function is applied at the level 1 predictions. Thus, $P_{final}=majority\left(\left\{P_{1}^{T},P_{2}^{T},P_{3}^{T},........,P_{p}^{T}\right\}\right)$. It is important to note that recently published studies have shown that different types of voice data are sensitive to different types of features and classifiers (Ali et al., 2019d; Ali and Bukhari, 2020; Gao et al., 2020a,b; Ahmad et al., 2021). Hence, based on these findings, for each data modality, a specific type of feature set and classifier was utilized at the base level.

In case of blending approach, a meta-classifier denoted by *C*_*M*_ is developed. To train the *C*_*M*_ model, the level 1 predictions, i.e., *P* = {*P*_1_, *P*_2_, *P*_3_, ........, *P*_*p*_} are modeled as input features of *C*_*M*_. After training the *C*_*M*_ model, it is tested using the testing data. During testing phase, the testing data (original features of the database) are given to the trained base classifiers, which will yield a set of prediction, i.e., $P^{T}=\left\{P_{1}^{T},P_{2}^{T},P_{3}^{T},........,P_{p}^{T}\right\}$. The set of prediction acts as set of features (input) for the *C*_*M*_ model. Thus, the meta classifier will produce final predictions for the testing data. These predictions are compared with the ground truth values, i.e., true labels of the data and the PD detection accuracy is obtained. In order to construct an optimal blending model, it is important to explore the feasibility of different machine learning models as the *C*_*M*_ model. In this study, we evaluated the feasibility of five renowned machine learning models, namely Linear Discriminant Analysis (LDA), Gaussian Naive Bayes (GNB), K-Nearest Neighbors (KNN), Support Vector Machine (SVM), and Artificial Neural Network (ANN) as the *C*_*M*_ model. The working of the proposed MMDD-Ensemble is more clearly described in Figure 1.

**Algorithm 1:** Multimodal Data–Driven Ensemble (MMDD-Ensemble) approach.

**Input:** {Multimodal Data Modalities *D*_1_, *D*_2_, ..., *D*_*i*_, ....., *D*_*m*_, Feature Sets *F*_1_, *F*_2_, ..., *F*_*j*_, ....., *F*_*n*_ and Classifiers *C*_1_, *C*_2_, ...., *C*_*k*_, ....., *C*_*p*_ }
**Output:** {*P*_*final*_, i.e., predictions by the MMDD-Ensemble }
1. Preprocessing multimodal data *D*_1_, *D*_2_, *D*_3_, ....., *D*_*m*_ and Feature sets *F*_1_, *F*_2_, *F*_3_, ....., *F*_*n*_
2. Develop ML models *C*_1_, *C*_2_, *C*_3_, ....., *C*_*p*_
3. Monitor $Acc=\frac{TP+TN}{TP+TN+FP+FN}$ for different *D*_*i*_ under *F*_*j*_ and *C*_*k*_
4. Discard *D*_*i*_ with poor *Acc*
5. Perform data partitioning: $D^{Tr}=\left\{D_{1}^{Tr},D_{2}^{Tr},D_{3}^{Tr},........,D_{o}^{Tr}\right\}$ and $D^{T}=\left\{D_{1}^{T},D_{2}^{T},D_{3}^{T},........,D_{o}^{T}\right\}$, $D_{i}^{Tr}$: Training dataset of the *i*th modality, $D_{i}^{T}$: Testing dataset of the *i*th modality. *o*: optimal number of modalities.
6. Fit base classifiers: *m*_*i*_ = fit $C_{i}\left(D_{i}^{Tr},F_{i},Y_{i}\right)$. *m*_*i*_: *i*th trained classifier, *Y*_*i*_: labels of the *i*th modality training samples.
7. *P*_*i*_ = $m_{i}\left(D_{i}^{Tr}\right)$
8. $P_{i}^{T}$ = $m_{i}\left(D_{i}^{T}\right)$
9. Evaluate final predictions: Method 1: Voting *P*_*final*_ = majority$\left(\left\{P_{1}^{T},P_{2}^{T},P_{3}^{T},........,P_{p}^{T}\right\}\right)$
10. Method 2: Blending *C*_*M*_ = fit(*P, Y*) where C_*M*_: Metaclassifier. *P*: Predictions of base classifiers on training data, *Y*: Training data labels. *P*_*final*_ = CM(*D*^*T*^)

## 3. Validation and Evaluation

To validate the performance of different methods, in this paper we utilized train-test holdout validation approach. Following the approach of Almeida et al. (2019), the dataset is divided into two parts, i.e., training and testing parts, 75% of the data is used for training the above-mentioned machine learning models and the 25% of the holdout data is used for testing the trained models. For evaluation of the constructed models, classification accuracy (CA), specificity (*S*_*p*_), sensitivity (*S*_*n*_), and Mathews Correlation Coefficient (*MCC*) are brought into account. These parameters are formulated using variables like true positives (a), true negatives (b), false positives (c), and false negatives (d).


(1) $$\begin{array}{r} CA=\frac{a+b}{a+b+c+d} \end{array}$$

(2) $$\begin{array}{r} Sn=\frac{a}{a+d} \end{array}$$

(3) $$\begin{array}{r} Sp=\frac{b}{b+c} \end{array}$$

(4) $$\begin{array}{r} MCC=\frac{a\times b-c\times d}{\sqrt{\left(a+c\right)\left(a+d\right)\left(b+c\right)\left(b+d\right)}} \end{array}$$

## 4. Experimental Results and Discussion

In order to evaluate the effectiveness of the proposed multimodal-based framework and to compare it against the best unimodal frameworks, we utilized receiver operating characteristics (ROC) curves and area under the curve (AUC) along with the above-discussed evaluation measures. All the experiments were simulated using Python software package and scikit-learn library (Pedregosa et al., 2011).

### 4.1. Experimental Results for Unimodal Data Obtained Through SP Channel

In this section, we perform experiments using the four unimodal datasets obtained through SP channel. The main objective of this experiment is to explore the optimal data modality and optimal feature set in terms of PD detection. In this experiment, we developed five different machine learning models namely LDA, GNB, KNN, SVM, and ANN. The results in terms of PD detection accuracy by each of the five developed model for the S modality are tabulated in Table 2. The best accuracy of 88% is obtained using GNB model and YAFFE features.

**Table 2:** Performance of different models at different extracted features for speech (S) modality obtained from SP channel.

**Feat. set**	**SVM**	**GNB**	**LDA**	**KNN**	**NN**
1	64	56	72	68	64
2	64	56	76	68	64
3	64	56	76	64	60
4	64	76	76	68	36
5	66	88	68	64	36
6	64	60	72	64	32
7	64	72	48	64	36
8	64	84	72	64	40
9	64	76	76	64	56
10	64	60	80	64	44
11	64	40	76	64	36
12	64	40	76	64	36
13	64	40	84	64	68
14	64	60	60	72	36
15	64	60	64	64	36
16	64	56	80	64	28
17	64	84	84	64	48

The same five machine learning models were also developed for the phonation (P), unvoiced (U), and voiced (V) modalities collected through the SP channel. The results for the V, U, and P modalities are tabulated in Tables 3–5, respectively. For the U modality, best accuracy of 88% is produced by the LDA model. For the P and V modalities, LDA model yields 74.66 and 80% accuracy, respectively.

**Table 3:** Performance of different models at different extracted features for voiced (V) modality obtained from SP channel.

**Feat. set**	**SVM**	**GNB**	**LDA**	**KNN**	**NN**
1	64	64	80	64	64
2	64	64	80	64	44
3	64	56	72	60	48
4	64	72	80	72	36
5	64	72	80	64	36
6	64	52	72	64	44
7	64	68	72	64	36
8	64	76	76	60	36
9	64	72	80	64	48
10	64	36	80	64	64
11	64	64	72	64	40
12	64	64	64	64	48
13	64	40	76	64	68
14	64	52	72	60	36
15	64	64	68	64	68
16	64	56	64	64	36
17	64	36	80	68	36

**Table 4:** Performance of different models at different extracted features for unvoiced (U) modality obtained from SP channel.

**Feat. set**	**SVM**	**GNB**	**LDA**	**KNN**	**NN**
1	64	60	72	64	64
2	64	60	72	64	64
3	64	48	60	56	64
4	64	68	88	60	64
5	64	64	64	64	32
6	64	64	64	68	68
7	64	48	60	60	36
8	64	64	68	64	60
9	64	68	68	52	32
10	64	68	64	64	56
11	64	44	48	64	36
12	64	48	60	64	56
13	64	44	60	64	44
14	64	56	64	76	48
15	64	48	80	64	36
16	60	52	76	60	40
17	64	40	76	64	32

**Table 5:** Performance of different models at different extracted features for phonation (P) modality obtained from SP channel.

**Feat. set**	**SVM**	**GNB**	**LDA**	**KNN**	**NN**
1	65.33	64	61.33	68	61.33
2	65.33	64	73.33	68	65.33
3	65.33	50.66	74.66	61.33	52
4	65.33	65.33	72	65.33	34.66
5	65.33	62.66	64	65.33	34.66
6	65.33	42.66	74.66	65.33	42.66
7	65.33	56	68	69.33	61.33
8	65.33	58.66	69.33	64	57.33
9	65.33	62.66	73.33	64	68
10	65.33	40	65.33	66.66	57.33
11	65.33	38.66	64	65.33	36
12	65.33	38.66	58.66	64	38.66
13	65.33	38.66	68	65.33	65.33
14	65.33	45.33	73.33	65.33	34.66
15	65.33	65.33	57.33	65.33	34.66
16	65.33	69.33	57.33	68	32
17	65.33	66.66	64	64	64

### 4.2. Experimental Results for Unimodal Data Obtained Through AC Channel

In this section, we perform experiments using the four unimodal datasets collected through the SP channel. The main objective of this experiment is to explore the optimal data modality and optimal feature set that would provide better PD detection accuracy for the data collected through AC channel. Again, we developed the five machine learning models, i.e., LDA, GNB, KNN, SVM, and ANN for each data modality. The results in terms of PD detection accuracy by each of the five developed models for the S modality are tabulated in Table 6. The best accuracy of 84% is obtained using the LDA model.

**Table 6:** Performance of different models at different extracted features for speech (S) modality obtained from AC channel.

**Feat. set**	**SVM**	**GNB**	**LDA**	**KNN**	**NN**
1	64	68	68	64	40
2	64	68	76	64	64
3	64	68	76	64	56
4	64	80	76	60	36
5	64	68	76	64	36
6	64	60	72	80	48
7	64	56	72	64	36
8	64	72	76	64	36
9	64	64	76	64	36
10	64	40	72	64	64
11	64	44	76	64	36
12	64	40	76	64	36
13	64	40	76	64	64
14	64	44	72	52	36
15	64	72	68	64	36
16	64	40	76	64	32
17	64	76	84	64	64

The above-discussed machine learning models were also developed for the phonation (P), unvoiced (U), and voiced (V) modalities collected through the AC channel. The results for the U, P, and V modalities are tabulated in Tables 7–9, respectively. For the U modality, best accuracy of 83.33% is produced by the LDA model. For the P and V modalities, LDA model yields 84% accuracy.

**Table 7:** Performance of different models at different extracted features for voiced (V) modality obtained from AC channel.

**Feat. set**	**SVM**	**GNB**	**LDA**	**KNN**	**NN**
1	64	68	56	64	64
2	64	68	72	64	64
3	64	68	64	64	60
4	64	72	68	60	36
5	64	68	60	64	36
6	64	68	68	64	44
7	64	64	52	64	36
8	64	72	68	76	36
9	64	68	76	64	36
10	64	68	36	68	56
11	64	68	60	68	36
12	64	68	56	68	36
13	64	32	48	68	64
14	64	40	68	52	36
15	64	64	56	48	52
16	58.3	54.1	62.5	58.33	50
17	64	64	84	72	36

**Table 8:** Performance of different models at different extracted features for unvoiced (U) modality obtained from AC channel.

**Feat. set**	**SVM**	**GNB**	**LDA**	**KNN**	**NN**
1	64	40	64	64	64
2	64	40	44	64	64
3	64	76	68	68	68
4	64	64	72	64	40
5	64	68	76	60	40
6	64	72	68	76	56
7	64	60	72	56	36
8	64	64	72	60	68
9	64	72	80	44	36
10	64	36	60	64	64
11	64	36	60	64	36
12	64	36	68	64	52
13	64	36	76	64	64
14	64	56	68	60	40
15	64	64	60	68	36
16	62.5	54	83.33	62.5	41.66
17	64	36	76	56	64

**Table 9:** Performance of different models at different extracted features for phonation (P) podality obtained from AC channel.

**Feat. set**	**SVM**	**GNB**	**LDA**	**KNN**	**NN**
1	65.33	65.33	66.66	64	65.33
2	65.33	65.33	66.66	64	66.66
3	65.33	44	74.66	56	60
4	65.33	56	73.33	66.66	62.66
5	65.33	57.33	72	66.66	37.33
6	65.33	40	76	70.66	52
7	65.33	48	58.66	65.33	61.33
8	65.33	58.66	70.66	66.66	53.33
9	65.33	53.33	64	66.66	50.66
10	65.33	64	62.66	66.66	60
11	65.33	64	69.33	60	34.66
12	65.33	64	68	60	37.33
13	65.33	64	64	65.33	40
14	65.33	60	74.66	70.66	34.66
15	65.33	72	66.66	65.33	65.33
16	65.33	68	58.66	68	32
17	65.33	74.66	84	69.33	34.66

### 4.3. Evaluation Measures for the Best Unimodal Results

In this section, we calculate the different evaluation measures discussed above for the best results obtained under the conventional unimodal approach. These results for the unimodal data of both the channels, i.e., AC and SP are tabulated in Table 10. It can be seen from the table that for the AC channel the best performance offered by the phonation modality and voiced speech modality is 84% of PD detection accuracy. On the other hand, for the SP channel the best performance offered by the phonation modality and voiced speech modality is 88% of PD detection accuracy. From the results of unimodal data, it is evident that better PD detection is obtained using YAFFE features for the AC data and for SP data, better results are produced by EM features.

**Table 10:** Best performance offered by individual modalities.

**Modality**	**Channel**	**Feat. set**	**Model**	**Acc (%)**	**Sen (%)**	**Spec. (%)**	**MCC**
P	AC	17	LDA	84.00	87.75	76.92	0.646
S	AC	17	LDA	84.00	93.75	66.66	0.645
U	AC	16	LDA	83.33	86.66	70.00	0.644
V	AC	17	LDA	84.00	100	55.55	0.666
P	SP	3	LDA	74.66	71.42	80.76	0.497
S	SP	5	GNB	88.88	93.75	77.77	0.736
U	SP	4	LDA	88.88	93.75	77.77	0.736
V	SP	4	LDA	80.00	81.25	77.77	0.578

*Sensitivity; Spec. (%), Specificity. Feat. Set, The type of feature set used; AC, Acoustic Cardioid; SP, Smart Phone; Acc(%), Classification accuracy; Sen. (%)*.

### 4.4. Results Produced by the Proposed MMDD-Ensemble Approach

#### 4.4.1. Experimental Results Produced by Fusing the Multimodalities of AC Channel Through the Proposed Approach

In this experiment, fusion of the multimodalities collected through the AC channel is carried out by using two different approaches, i.e., voting and blending. The experimental results are given in Table 11. Under the voting criterion, optimal performance of 88% of PD detection accuracy is obtained while the blending approach produced 92% of PD detection accuracy. By comparing the results offered by the proposed multimodal approach with the best results offered by optimal unimodal data, it is evident that the proposed approach improves PD detection accuracy by 8% for the data collected through AC channel.

**Table 11:** Performance offered by the proposed multimodal approach using AC channel.

**Fused modalities**	**Method**	**M.Model**	**Acc (%)**	**Sen (%)**	**Spec. (%)**	**MCC**
V+S	Voting	-	84	100	55.55	0.666
V+U+S	Voting	-	84	100	55.55	0.666
U+S+P	Voting	-	80	100	44.44	0.581
V+U+S+P	Voting	-	88	100	66.66	0.749
V+S	Blending	KNN	84	100	55.55	0.666
V+U	Blending	KNN	92	100	77.77	0.831
V+U+S	Blending	GNB	92	100	77.77	0.831
V+U+S+P	Blending	GNB	92	100	77.77	0.831

*Method, Fusion method; M. Model, Meta Model; Acc(%), Classification accuracy; Sen. (%), Sensitivity; Spec. (%), Specificity*.

**Figure 2:**
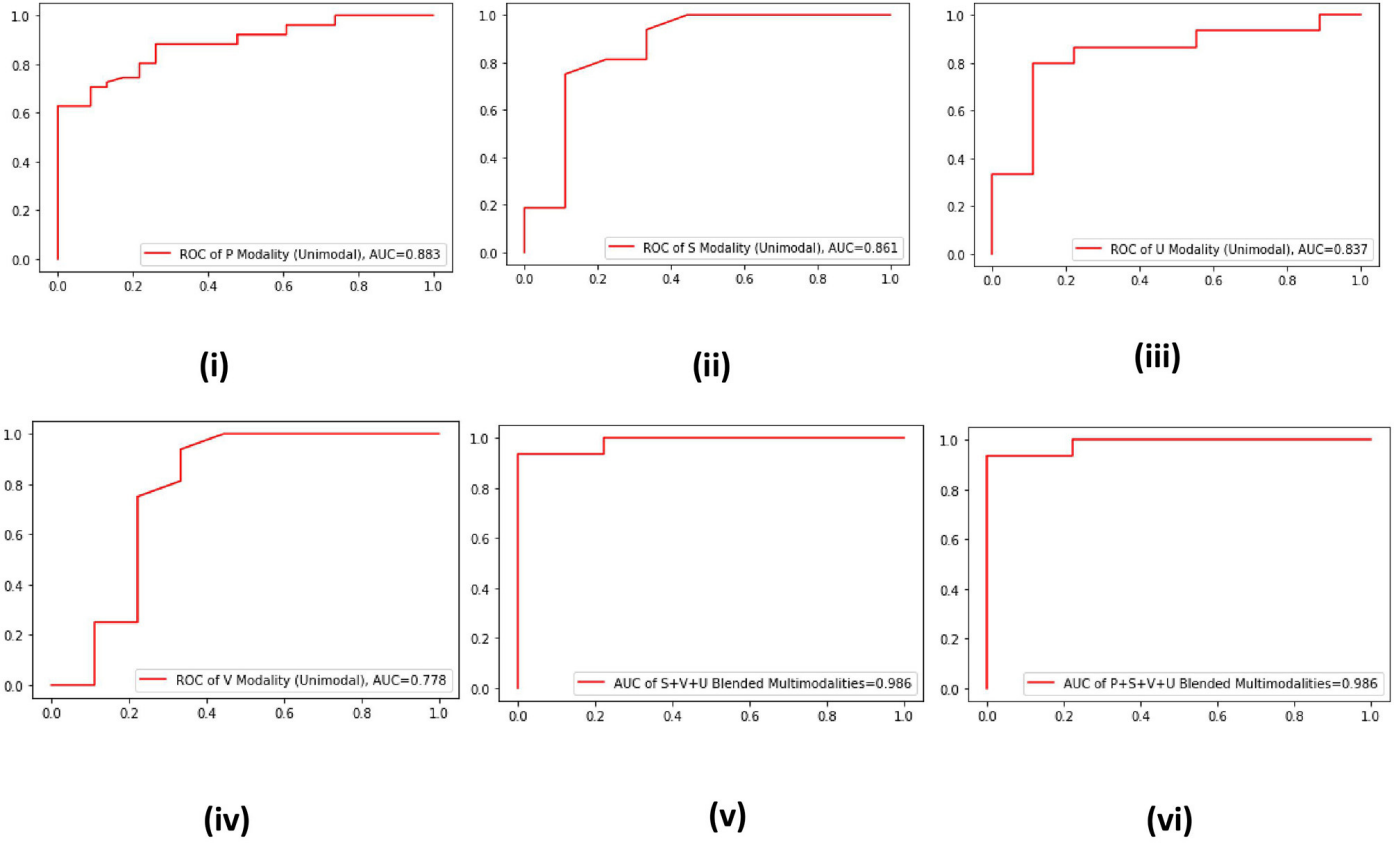
**(i–vi)** Receiver operating characteristics (ROC) curves of four unimodal AC channel data–driven systems and two multimodal data–driven blended systems.

In machine learning, ROC curve is a more robust evaluation metric that is used to check the robustness of a developed model against baseline models. A model having an ROC curve with more AUC is declared robust compared to models having ROC with less AUC. Hence, to validate the effectiveness of the proposed multimodal approach, we plot the ROC curves of the four unimodal and two multimodal systems for the data of AC channel (Figure 2). It is evident from the ROC curves that the best AUC is offered by the P modality of AC channel, which is 0.883 (Figure 2i). On the other hand, an AUC of 0.986 is produced by two blended multimodalities, i.e., P+S+U+V and S+V+U (Figures 2v,vi). Hence, the effectiveness of the proposed multimodal approach is validated from both aspects, i.e., accuracy and AUC.

#### 4.4.2. Experimental Results Produced by Fusing the Multimodalities of SP Channel Through the Proposed Approach

In this experiment, fusion of the multimodalities collected through the SP channel is carried out. Again, two different approaches, i.e., voting and blending were adopted while fusing the multiple types of modalities. The experimental results are given in Table 12. Both the voting and blending criteria yielded PD detection accuracy of 96%. However, the best PD accuracy with unimodal approach is 88%. By comparing the results offered by the proposed multimodal approach with the best results offered by optimal unimodal data, it is evident that the proposed approach improves PD detection accuracy by 8% for the data collected through AC channel.

**Table 12:** Performance offered by the proposed multimodal approach using SP channel.

**Fused modalities**	**Method**	**M. Model**	**Acc (%)**	**Sen (%)**	**Spec. (%)**	**MCC**
V+S	Voting	-	92	100	77.77	0.831
V+U+S	Voting	-	96	100	88.88	0.941
S+P	Voting	-	92	93.75	88.88	0.826
V+U+S+P	Voting	-	96	100	88.88	0.941
V+S	Blending	ANN	92	93.75	88.88	0.826
V+U+S	Blending	ANN	96	100	88.88	0.914
V+U+S+P	Blending	ANN	96	100	88.88	0.914

*Method, Fusion method; M. Model, Meta Model; Acc(%), Classification accuracy; Sen. (%), Sensitivity; Spec. (%), Specificity*.

**Figure 3:**
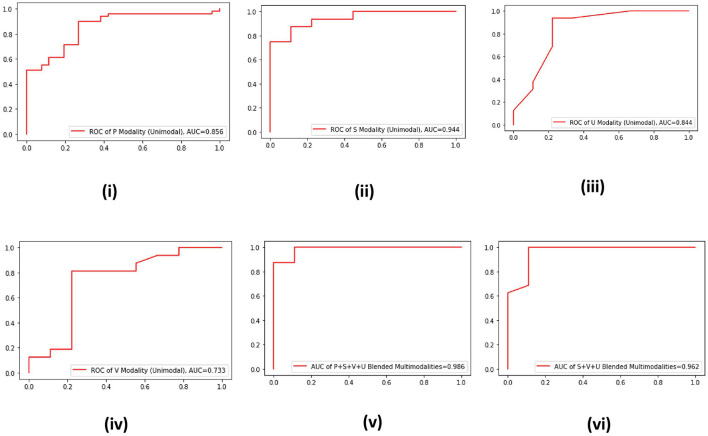
**(i–vi)** ROC curves of four unimodal13 SP channel data-driven systems and two multimodal data–driven blended systems.

For the data collected through the SP channel, to validate the effectiveness of the proposed multimodal approach, we plot the ROC curves of the four unimodal data–driven systems and two multimodal data–driven systems (Figure 3). It is evident from the ROC curves that the best AUC is offered by the S modality of SP channel, which is 0.944 (Figure 3ii). On the other hand, an AUC of 0.986 is produced by blended multimodalities, i.e., P+S+U+V and AUC of 0.962 by the blended multimodalities S+V+U (Figures 2v,vi). Hence, the effectiveness of the proposed multimodal approach is also validated for the SP channel data.

### 4.5. Comparative Study With State-Of-The-Art Ensemble Learning Models and Recently Published Work

In order to further validate the effectiveness of the proposed multimodal approach, a comparative study is conducted with recently published work (given in Table 13) and with other state-of-the-art ensemble learning models. The renowned ensemble machine learning models, namely Adaboost ensemble model, Random Forest (RF) ensemble model, and Gradient Boosting Ensemble model, were developed. The Adaboost ensemble model produced optimal performance of 89.33% accuracy and AUC of 0.936 for the P modality of the AC channel. The Gradient Boosting model achieved 86.48% accuracy and 0.921 of AUC. Finally, the RF model resulted in 88% of accuracy and 0.910 of AUC for the S modality of the SP channel. After comparing and analyzing the results of the proposed MMDD-Ensemble approach and other ensemble learning approaches, it is clear that the proposed approach yields better results. Although the proposed approach uses simple fusion approaches, it still enhances the performance. The main reason for yielding improved results is that the different base classifiers of the MMDD-Ensemble method are driven by different types of optimal data modality. Hence, the optimal results of unimodal data are fused or ensembled, consequently the final results are better than the optimal unimodal results. On the other hand, conventional ensemble methods (i.e., Adaboost, RF etc) are unimodal data–driven approaches; hence, their results are comparable with optimal unimodal results but poor compared with the results of the MMDD-Ensemble method.

**Table 13:** Comparative study with recently published work.

**Study/Model (year)**	**Method**	**Accuracy (%)**	**AUC**
Adaboost Ensemble	MP+Adaboost+AC	89.33	0.936
Random Forest	TS+Random Forest+SP	88.00	0.910
Gradient Boosting Ensemble	YA+Gradient Boost+AC	86.48	0.921
Vaiciukynas et al. (2017)	TS+Random Forest+SP	79.00	–
Vaiciukynas et al. (2017)	IS5+Random Forest+AC	62.40	–
Almeida et al. (2019)	IS6+SVM+AC	76.00	0.780
Almeida et al. (2019)	MPEG7+KNN+SP	72.00	0.740
Almeida et al. (2019)	KTU+KNN+SP	94.00	0.870
Almeida et al. (2019)	YAFFE+KNN+AC	92.00	0.920
This study (2020)	Multimodal ensemble approach	96.00	0.986

## 5. Conclusion and Future Studies

In this paper, PD detection based on multimodal voice and speech data was considered. Data were collected from two channels, i.e., AC and SP. After developing and exploring performance of different machine learning models, it was observed that the best PD detection accuracy of 84% and 88% is obtained under unimodal approach for the AC and SP channels data, respectively. In order to improve the PD detection accuracy, we developed an MMDD-Ensemble approach. The proposed approach produced PD detection accuracy of 92% and 96% for the AC and SP channel data, respectively. Thus, it was pointed out the proposed multimodel approach outperformed the best results offered by optimal unimodal approach. Moreover, the proposed method showed better results than other renowned state-of-the-art ensemble models and previously reported methods. On the basis of experimental results, the effectiveness of the proposed multimodal approach was validated.

The proposed MMDD-Ensemble approach yielded better performance; however, the fusion approaches used are simple. Therefore, in future studies, some more advanced fusion methods like graph fusion (Mai et al., 2020) and tensor fusion (Zadeh et al., 2017) can also be explored. Additionally, in future, the focus should be on collection of large scale datasets and deep neural network based base classifiers.

## Data Availability Statement

Publicly available datasets were analyzed in this study. This data can be found here: https://journals.plos.org/plosone/article?id=10.1371/journal.pone.0185613#sec021.

## Ethics Statement

Ethical review and approval were not required for the study on human participants in accordance with the local legislation and institutional requirements. Written informed consent from the participants or participants' legal guardian/next of kin was not required in this study in accordance with the national legislation and the institutional requirements.

## Author Contributions

LA: conceptualization, formal analysis, methodology, software, validation, writing-original draft, and writing—review and editing. ZH and WC: investigation, software, resources, supervision, and writing—review and editing. HR, YI, and MB: formal analysis, methodology, validation, visualization, and writing—original draft. All authors contributed to the article and approved the submitted version.

## Funding

This work was supported by National Natural Science Foundation of China under grants 61971290, 61771322, and 61871186 and the Fundamental Research Foundation of Shenzhen under grant JCYJ20190808160815125.

## Conflict of Interest

The authors declare that the research was conducted in the absence of any commercial or financial relationships that could be construed as a potential conflict of interest.

## Publisher's Note

All claims expressed in this article are solely those of the authors and do not necessarily represent those of their affiliated organizations, or those of the publisher, the editors and the reviewers. Any product that may be evaluated in this article, or claim that may be made by its manufacturer, is not guaranteed or endorsed by the publisher.
